# The Work of the County Medical Society

**Published:** 1911-05

**Authors:** 


### The Work of the County Medical Society

THE anaphylaxis of our public schools and the enormous morbidity of our children are facts of prime significance. We believe our city to be most fortunate in having a problem of such importance receive the earnest attention of bodies as representative and influential as are the Buffalo Physical Education Society and the Medical Society of the County of Erie. These two societies recently gave this subject particular consideration and made prominent the following important facts.

Within the three years last past the morbidity from scarlet fever, diphtheria, measles and whooping cough has been about twenty-five thousand cases; the morbidity from other of the preventable diseases has also been high. The last annual report of the Commissioner of Health of Buffalo, showed more than twelve thousand defective children in our public schools. It is safe to infer there are nearly as many more in private schools where nearly half of our children are being taught. Our public school authorities inform us that upon a recent survey of the subject it was found of the then pupils, more than seventeen thousand were or had been repeaters and that the number of their repetitions was more than thirty thousand.

The official records of the health department show that the ventilation of public schools when last tested was most unsatisfactory, discreditable and anaphylactic; that in all of the schools examined, lack of uniformity of air supply in class rooms was present to a scandalous degree; that in an overwhelming majority of all the schools examined the amount of air furnished was from one-third to two-thirds less than the quantity required to maintain good health of the children and teachers; that tests of the ventilation by measurements of the foul air taken out of the class rooms of all the schools examined revealed the revolting condition that the promises of the builders were made good in about 1 per cent. of the rooms and that the actual work of ventilation was from one-half to one-tenth of that named in the contract and paid for by the city; that the presence of $CO_2$, the index of defilement of the air, was found almost invariably to be dangerously high and in too many instances to be five and six and seven times that which may be breathed in safety.

Notwithstanding this official demonstration of the incompetency of the prevailing system of ventilation and in spite of the fact that all recent contracts for school buildings call for ventilating apparatus that will not only promise, but actually perform and that in precise and intelligent amounts, we find that seventeen schools have been built with systems of ventilation similar to those tested in brazen defiance of the terms of the contracts and, moreover, all have been paid for in full and not one has been tested.

We were also informed by an experienced teacher from Chicago that the open air school is no longer an experiment but by demonstration is now a scientific phase of school life, a scientific answer and remedy for the anaphylaxis of school air.

Pupils are more comfortable, more studious, more receptive, more healthful in an atmosphere free from respiratory excrement and properly humid, with a temperature from 10 to 20 per cent. lower than the usual class room temperature. The educational, economic and hygienic significance of this practical observation is obvious. Responsibility is measured by opportunity and opportunity is spaced by knowledge; and knowledge of the communicable diseases is increasing daily.

The responsibility of the three-sided situation, serious and important from each and every side—hygienic, educational and economic—is not resting now upon the members of the medical profession or the medical society. What action will be taken by those in authority? We shall watch with interest.

